# Carrying the Burden Into the Pandemic – Effects of Social Disparities on Elementary Students’ Parents’ Perception of Supporting Abilities and Emotional Stress During the COVID-19 Lockdown

**DOI:** 10.3389/fpsyg.2021.750605

**Published:** 2022-01-11

**Authors:** Markus Vogelbacher, Manja Attig

**Affiliations:** Competencies, Personality, Learning Environments, Leibniz Institute for Educational Trajectories, Bamberg, Germany

**Keywords:** parental stress, parental support, COVID-19 pandemic, social disparities, parents of second graders

## Abstract

The COVID-19 pandemic has posed many challenges, especially for families. Both the public and the scientific community are currently discussing the extent to which school closings have worsened existing social differences, especially with regard to children’s academic and socio-emotional development. At the same time, parents have had to manage childcare and home schooling alongside their jobs and personal burdens posed by the pandemic. Parents’ possibilities for meeting these cognitive and emotional challenges might also depend on the different conditions in families. For this reason, the present paper investigates the structural and process characteristics of the family as well as children’s and parents’ psychological characteristics that predict how parents assess their ability to support their child’s learning during homeschooling as well as parents’ perceived emotional stress caused by school closure. The study analyses data of the Newborn Cohort Study of the German National Educational Panel Study. The two dependent variables (self-assessment of abilities, perceived stress) were measured during the COVID-19 pandemic after the first school closure in Germany, at a time when the children of this cohort were attending second grade. Besides a number of control variables (including the child’s struggle with home schooling), families’ structural characteristics [socioeconomic status (SES), education], process characteristics (home learning environment, HLE), parents’ psychological characteristics (preceding psychological stress), and the child’s psychological characteristics (self-regulation, school-related independence) from earlier waves were included as predictors. The results of structural equation models show that perceived stress was associated with structural factors and the preceding psychological stress of parents. Parents with higher preceding stress reported higher perceived stress. Interestingly, higher-educated parents also reported more stress than lower educated parents during the pandemic. The effect was the other way around for SES – parents with lower SES reported more stress than parents with higher SES. The self-reported abilities to support the learning of the child seemed to be mainly predicted by the parent’s education as well as preceding psychological stress. To sum up, the results identify important aspects that determine how parents handle the challenges of the school closures. Especially, socially disadvantaged families carry their burden into the pandemic.

## Introduction

In spring 2020, the COVID-19 pandemic swept around the world, bringing about many changes in the daily lives of people. To limit the spreading of the pandemic, many countries decided to send their inhabitants into a lockdown, leading to drastic changes that affected family, personal and work life. The lockdowns often prescribed closures of schools and other childcare facilities, presenting challenges to families. Already in the first wave of the pandemic, schools were closed in Germany. Hence, schoolchildren had to be taught at home from March 13, 2020 until the first schools re-opened in May. During this time, all institutional and non-institutional childcare offers were closed (for an overview regarding the situation in Germany, see Helm et al., 2021). From May or June, depending on the federal state, until the start of the summer breaks, the primary schools were not back to regular schooling, but implemented various measures to reduce social contacts in schools, e.g., changing classes with fewer students in the schools. Consequently, homeschooling remained a part of the families’ lives until the start of the holidays at the end of June to the end of July.

As a result, parents had to deal with multiple challenges such as homeschooling (providing technical and learning support), the reorganization of childcare and their own dynamic working situation. In addition, they were confronted with social isolation and changes in their personal lives. Furthermore, this uncertain pandemic situation affecting many life domains caused emotional strain which people had to deal with (see e.g., Cheng et al., 2021).

Depending on the school age of their children, parents were faced with different challenges. Whereas older students were more able to learn on their own and deal with the technical aspects of home schooling and distance learning, younger students needed more support with learning at home. Further, the parents of younger students also had to deal with childcare in addition to supporting and monitoring the learning of their children. Before the lockdown, around half of the elementary school students in Germany attended additional childcare facilities and all-day schools (Autorengruppe Bildungsberichterstattung, 2020). These childcare facilities were closed during the pandemic at the expense of parents. In addition, elementary school students are less experienced in working with digital media than older students (see Schmid et al., 2017).

To sum up, parents were confronted with considerable challenges. They needed time and ability to offer school-related help and handle the mental strain posed by the lockdown situation. How parents dealt with the challenges of the pandemic and school closures, depended on the family situation, their personal resources, and the process characteristics of the HLE (Prime et al., 2020). Hence, parents experienced the pandemic in very different conditions. In addition, parents’ ability to provide support to their children and their own wellbeing or stress can be assumed to be interdependent (Kohl et al., 2000; Yeung et al., 2002; Baranov et al., 2020).

During the course of the pandemic, discussions in the public and academic community shifted toward the negative academic and socio-emotional consequences on children due to the absence of professional teaching in an institutional setting and the loss of social contacts (Loades et al., 2020; Kaffenberger, 2021). These consequences were suspected to vary strongly as a result of social inequality, with the public and researchers aware of the danger that existing inequalities would be exacerbated by the pandemic (Eickelmann and Drossel, 2020; Huebener et al., 2020; Wößmann et al., 2020). In a similar vein, differences in the availability and use of technological resources in families were also likely to deepen social inequalities (Sari et al., 2021).

The discussion of the problematic cognitive and socio-emotional effects of school closures mainly focused on children (Loades et al., 2020; Kaffenberger, 2021). The challenges posed on parents by school closures, on the other hand, were often brought up in the context of the operational compatibility of homeschooling, the frequently changing working situations, and lack of childcare offers (Möhring et al., 2020; Müller et al., 2020).

As parents’ emotional and cognitive ability to deal with the challenges of home learning is a most important determinant of the consequences of school closures on children (see e.g., Spinelli et al., 2020), it seems worthwhile to investigate the conditions affecting their emotional and cognitive potential to face the pandemic. Identifying these conditions is not only of academic interest, but a requirement in offering appropriate practical support to families challenged by the school closures. Hence, the aim of the present paper is to investigate the emotional as well as the cognitive dealing of parents of elementary students as these parents had to manage childcare and support the learning of their children. As the conditions differed between families already before the pandemic (e.g., Hart and Risley, 1995; Bradbury et al., 2015), a longitudinal view was needed to take pre-existing differences into account. To do so, the present paper analyzed longitudinal data. As social inequalities are assumed to have increased, one main aim of the present paper was to analyze whether preceding social differences influenced how parents dealt with the challenges of the pandemic, focusing on their emotional stress and their ability to support the learning of their child at home.

## Theoretical and Empirical Background

### The Role of Parental Support and Parental Stress in the Homeschooling Situation

Recent empirical research on children’s dealing with homeschooling showed that some children have troubles adjusting to the unfamiliar situation of learning at home (e.g., structuring their day, their tasks and to motivate themselves to learn) without professional guidance (Champeaux et al., 2020; Davis et al., 2020; Huber et al., 2020). Unlike regular school days, parents in homeschooling have a much bigger responsibility for ensuring that their children learn the subject matter (Greenhow et al., 2021). This applies especially for parents of younger school children. Supporting children requires parents to have both the ability to understand the school material and the didactic skills to convey it. Furthermore, parental self-efficacy in teaching is found to be a predictor of parent-child conflict (de Jong et al., 2021). Consequently, we assume that parental support of learning is a focal point of children not losing touch with the learning content and a crucial predictor of their educational trajectories during the pandemic. In addition to the aspect of practical support with subject matter, the emotional support of the parents can be considered as an important determinant of children’s academic success in home learning (see, e.g., Mayo and Siraj, 2015). Emotional support, parental warmth and affective involvement are requirements of children’s cognitive and socioemotional development and depend on parents’ mental wellbeing (Conger et al., 1992; Wu et al., 2019), which comes under pressure in the pandemic (Spinelli et al., 2020). Even before the pandemic, qualitative research in U.S. families, who decided voluntarily to teach their children at home, described the integration of the teacher role in parent’s everyday life as emotionally challenging up to causing emotional burnout (Lois, 2006). Thus, we consider parents’ perceived stress as a determinant for the ability to face the challenges of homeschooling and provide emotional support to the child.

### Conceptual Framework on Risks to Families’ Wellbeing in the COVID-19 Pandemic

Already in the first lockdown of the pandemic, Prime et al. (2020) proposed a conceptual framework representing how the pandemic would potentially influence different parts of the family – the children, parents, and the family as a whole. Similar to bioecological models of development (e.g., Bronfenbrenner and Morris, 2006), the framework assumes that children are influenced by both distal factors and proximal processes in the pandemic. As central determinant of the family processes during the pandemic, the framework focuses on a cascading effect, starting from an unexpected increase of daily stressors directly connected to the pandemic, like financial insecurity and social distancing. These stressors exert a detrimental influence to the mental well-being of caregivers, which in turn shows harmful effects on the parent–child-interaction, the marital relationship and the family system as a whole. Further, the framework also suggests that pre-existing vulnerabilities (e.g., economic hardship, mental health) may influence how parents cope with the situation of the pandemic. The pandemic is assumed to have a bigger impact on families with a lower socioeconomic background (Prime et al., 2020).

### Family Stress Model and Family Investment Model

In a similar but more specific direction, parental emotional strain and the ability to offer school-related help to the child are crucial factors in two of the most prominent theoretical models that explain the links between the socioeconomic background of the family and disparate academic and socio-emotional developmental outcomes of the child: the Family Stress Model (FSM; Conger et al., 1992) and the Family Investment Model (FIM; Conger and Donnellan, 2007):

According to the FSM (Conger et al., 1992), socioeconomic disparities in the academic and socio-emotional development of children are mediated by parental emotional strain. The model assumes that strain is caused by economic scarcity and negative economic events which lead to parent’s depressive symptoms, an increase in marital conflicts, and in turn detrimental parenting behaviors, which influence children’s cognitive and socio-emotional development adversely. Abidin’s (1992) Parenting Stress Model focuses on parenting stress and extends the FSM by adding the crucial role of child characteristics which also can explain or influence parenting stress. Accordingly, children’s social skills and problem behavior predict parental mental strain (Abidin, 1995).

The FIM (Conger and Dogan, 2007; Conger and Donnellan, 2007) describes another pathway for the effect of families’ social background on children’s health and cognitive and socio-emotional development. Rooted in economic theory (Mayer, 1997), the FIM deals with the ability of the family to invest financial, social and human capital (e.g., involvement in the academic education of the child) in the development of the child. This ability depends on the allocation of resources in the family. While parents with a lower socioeconomic status (SES) need to invest most of their resources meeting immediate needs (e.g., housing, food, clothing), parents with a higher SES possess excess resources which allow them to invest more in their child’s development. According to Conger and Donnellan (2007), there are several mediators of the effects of financial, social, and human capital on child outcomes: standard of living; residence in a more or less protected and fostering environment; the provision of learning materials and the parent’s stimulation and support of learning, both directly and through external training. The latter is more specifically related to the parent’s involvement in the academic education of the child. Hence, parent’s ability to help his or her child with schoolwork can be seen as one aspect of parental investment (Simons et al., 2016) and has been found to be a strong predictor of children’s academic outcomes (Liu and Leighton, 2021). Kohl et al. (2000) suggest disentangling the SES into its components (income, occupation, parental education) when investigating its effects on parental involvement in children’s learning. Consequently, they solely examine the structural influence of parent’s education, for which they find significant fostering effects on parental involvement at home (but there are also studies pointing out the crucial role of the material family background in predicting the parental home involvement, see, e.g., Green et al., 2007).

Yeung et al. (2002) suggested that the potential pathways of structural effects on child outcomes should not be investigated in isolation. They propose a two-way model according to which emotional distress impairs parental engagement in stimulating learning activities, on the one hand, and previous parental investment in the HLE influences perceived mental strain, on the other. Yeung et al. (2002) only found empirical evidence for the beneficial effect of previous parental investment on parental stress. With data from a 16-year longitudinal study, Wu et al. (2019) reported mothers’ depression to decrease the provision of emotional and material learning support to their child. They also found low maternal support in middle childhood to predict higher levels of maternal depression later on. In addition, based on an intervention study on depressed mothers, Baranov et al. (2020) reported decreasing maternal depression to have long-term beneficial effects on parental investment as well. Taking a multi-dimensional approach on parental investment in children’s education, Kohl et al. (2000) differentiated parental involvement in school activities, exerting stimulating activities with the child and parent-teacher contact. They investigated risk factors for these dimensions of involvement and found, among other things, maternal depression to exert adverse effects on nearly all dimensions. This points to a potential reciprocal effect between the parent’s ability to provide support and his or her perceived stress, suggesting that an integrated analysis of the predicting factors for both parental outcomes is required.

To sum up, the theoretical models introduced above propose different (pre-existing) aspects that may influence how families and parents cope with the challenges of the pandemic and, in turn, influence how the child copes and develops. Following the models (Conger et al., 1992; Conger and Donnellan, 2007), it seems worthwhile, on the one hand, to investigate the influence of structural social differences on how parents dealt with the pandemic. On the other hand, potential mediators such as individual psychological and family process aspects (e.g., home learning environment/stimulating activities; Kluczniok et al., 2013) should be taken into account in the analysis of social differences.

### Family Stress and Family Investment During the Pandemic

In regard to both models, the FSM and the FIM (Conger et al., 1992; Conger and Donnellan, 2007), the COVID-19-pandemic can be conceptualized as an additional challenge to all families with children. However, the expected burden seems higher for disadvantaged families.

In the FSM, the COVID-19-pandemic and therefore the school closure can be modeled as a collective stressor (Zinn and Bayer, 2021), which presents a challenge to all parents. It could be assumed that the disparities in the available resources of families and the mental health of parents prior to the pandemic, however, result in parents perceiving the challenges accompanying the lockdown differently, leaving disadvantaged families mentally more worn out. For example, Zinn and Bayer (2021) found that lower-educated parents and single parents report a higher amount of stress during the pandemic. On top of that, parents with lower SES had a higher risk of being affected by short-time work measures or of losing their jobs during the pandemic and had fewer possibilities of working from home (Kleinert et al., 2020). Such negative economic events as well as the lack of health protection and possibility to supervise one’s child’s learning can function as additional stressors, further increasing the mental strain of parents (Calvano et al., 2021).

Recent empirical studies have investigated the predictors of parental experience of emotional demands such as stress, anxiety, and parent-child interaction in the context of the COVID-19 pandemic and found an influence of factors directly related to the pandemic situation (e.g., Brown et al., 2020; Davis et al., 2020; Cheng et al., 2021; Racine et al., 2021). For example, Brown et al. (2020) investigated the impact of the COVID-19 pandemic on parental perceived stress. They showed that – besides a higher number of COVID-19 related stressor – high anxiety and depressive symptoms were associated with the perceived stress of the parents. Further, Brown et al. (2020) used children’s problems with learning in homeschooling as an aspect of the cumulative scale of COVID-19 related stressors, which was associated with higher levels of perceived parental stress. Davis et al. (2020) examined the effects of homeschooling on parents’ mental health and corroborated the promoting role of children’s struggling with homeschooling in parents‘ anxiety and depressive symptoms. Cheng et al. (2021) reported that the necessity to care for their children during the lockdown led to an increase in mental health problems among working parents. In addition, the support provided to parents, for example, in the form of childcare, had a significant negative effect on depressive symptoms (Racine et al., 2021) and perceived stress (Brown et al., 2020) both of which decreased as support increased. This also holds true for the perceived support provided by schools (Porsch and Porsch, 2020). Furthermore, besides the impact of disruptive economic events, such as loss of income or employment (Cheng et al., 2021; Racine et al., 2021), the parent’s mental framing of the situation also had an effect: acceptance and perceived control of the situation were associated with lower perceived stress (Brown et al., 2020; Chung et al., 2020).

In addition to factors directly associated with the pandemic, the personal and structural conditions of parents and families prior to lockdown had an effect on the (perceived) stress during school closings (e.g., Brown et al., 2020). Based on self-reports, some cross-sectional studies (Brown et al., 2020; Chartier et al., 2021) found associations between perceived stress during the pandemic and depressive symptoms and levels of stress reported prior to the pandemic. Longitudinal data backing up this correlation are scarce (for longitudinal results on mental health, see Cheng et al., 2021; for longitudinal results with history of mental illness as predictor, see Racine et al., 2021). Moreover, demographic and structural factors impacted the perceived emotional stress during the pandemic. For example, Brown et al. (2020) found disparities in COVID-19-related stressors, such as parental mood and stress, among different ethnic groups, with members of minorities showing higher levels of stressors. Further, research has shown that women as well as younger parents and lower-educated parents experienced significantly higher levels of peritraumatic stress during the lockdown than men, older parents and higher-educated parents (Chartier et al., 2021). Going in a similar direction, Porsch and Porsch (2020) found stronger experiences of stress in female parents and in families with a higher numbers of school-aged children, but their results regarding the education of the parents were not in line with those in other studies. They reported that higher-educated parents experienced higher levels of stress. However, as they did not have any information on the preceding levels of stress and the previous family situation, a causal interpretation of this finding seems questionable. Porsch and Porsch (2020) also identified a negative association between the parental self-efficacy in supporting the child with school subject matter and the experience of stress and anxiety.

In the FIM (Conger and Dogan, 2007; Conger and Donnellan, 2007), parents’ obligations to accompany their child with his or her everyday learning and to carry out parts of school teaching themselves represent an investment need, which only higher-SES parents can fully meet. To provide adequate support to a child in a homeschooling situation, parents, relatives, and friends have to be able to understand and explain schoolwork, in addition to finding time to be involved in the child’s learning. If this ability to provide support is low, extra training and support for the child can be provided by external trainers, but this is commonly subject to payment of a fee.

Bol (2020) investigated parental ability to support their child with schoolwork during the lockdown using data from the Dutch LISS panel study. He found that parents’ subjective assessment of their ability to help their children with schoolwork was more positive for parents with higher educational degrees than parents with lower educational degrees. Furthermore, parental ability to support their child was identified as a crucial predictor for actual parental support. Socially privileged parents were already shown to have a higher ability to support their child before the COVID-19 pandemic and school closures (e.g., Anger and Plünnecke, 2020). These findings do not relate to the quantitative aspect of support. Whereas no social differences were found in the time spent supporting a child with homework (Lee and Bowen, 2006; Luplow and Smidt, 2019), effects were found for the quality of assistance (Dumont, 2012; O’Sullivan et al., 2014). Besides parental ability to provide help, the availability of material learning resources for homeschooling in a family (e.g., computers, tablets, wireless internet access) was associated with actual learning support (Bol, 2020; Sari et al., 2021). The availability of the necessary technological infrastructure for remote learning, for its part, depended on the economic resources of a family (Greenhow et al., 2021).

The previous empirical findings (e.g., Bol, 2020; Brown et al., 2020) support the assumption that different social and personal aspects play an important role in how parents deal with the pandemic. The conceptual framework (Prime et al., 2020), representing the impact of the pandemic on children, parents, and families, already proposed that not only aspects during the pandemic play a role, but also pre-existing vulnerabilities. Hence, the present paper aimed to investigate if pre-existing structural, process, and psychological characteristics of parents and children influence how parents deal emotionally and how they managed to support their child’s learning during the pandemic.

## Present Study

The present paper examines how parents of second-graders experienced the time during the first school closures in the COVID-19 pandemic in Germany, focusing on two aspects. The first aspect is the parent’s perception of their ability to support their child with home learning during the lockdown. The second aspect is the emotional stress experienced by parents during school closure. The aim of the paper is to determine which preceding structural and processual aspects of the family as well as psychological characteristics of the responding parent and the child helped parents to handle the challenge of navigating their own work, homeschooling, and childcare all at once. To answer the research questions, the present paper used longitudinal data collected in the Newborn Cohort Study of the National Educational Panel Study (NEPS). These data allow an analysis of how different aspects of family life before the pandemic help parents to handle the cognitive and emotional challenges during the school closure caused by the pandemic.

The following preceding aspects of family’s and personal characteristics were assumed to impact parents’ ability to provide support to their child as well as parents’ emotional stress during the lockdown as a result of the pandemic.

(1) Structural characteristics of the families, such as SES and education. Based on the predictions of the FSM (Conger et al., 1992), parents with a lower SES report more emotional stress than parents from a higher socioeconomic background. According to the FIM (Conger and Dogan, 2007; Conger and Donnellan, 2007) and current empirical research in the context of the COVID-19 pandemic (Bol, 2020), parents with lower levels of education assess themselves as less able to support the child with schoolwork.
(2) Process characteristics of the family, such as the home learning environment (HLE). We assumed that parents who were able and used to offering their child a stimulating HLE already before the pandemic, were better prepared for the extra investment in learning support and emotional challenges during school closure. We expected parents who reported a more stimulating learning environment before the pandemic to report that they were more able to provide support and have fewer emotional difficulties during the pandemic. Furthermore, we expected the HLE to function as a mediator of the structural effects on the outcomes during the pandemic.
(3) Psychological characteristics of the parent (focusing on the main caregiver), such as psychological stress. We expected parents with a higher level of psychological stress in the years before the pandemic to experience more problems in dealing emotionally with the pandemic as well as having more difficulties supporting their child’s learning.
(4) Psychological characteristics of the child, such as self-regulation as well as school-related independence. We expected parents of children with a higher level of self-regulation as well as school-related independence to report fewer difficulties during the pandemic, both emotional strain and difficulties in supporting the child.

## Materials and Methods

### Sample

The present paper used data from the Newborn Cohort Study of the NEPS (Blossfeld and Roßbach, 2019). This cohort study has a representatively drawn sample of around 3,500 infants born between February and June 2012 and their mothers (Weinert et al., 2016). Each year, the mother or another responding parent (since wave 2) take part in a computer-assisted parent interview. Further, in each wave the competencies of the children are assessed. Hence, the Newborn Cohort Study has both data from the years before the pandemic (waves 1–8) and data which collected during the pandemic (wave 9). Further, in wave 9 – in addition to the regular assessment – a short questionnaire regarding the COVID-19 pandemic was integrated into the survey in addition to the regular assessment. The present paper analyzes data from the cohort study waves 7–9 for children aged from 6 years (Kindergarten) to 8 years (Grade 2). The data from wave 9 were collected from June to August 2020, after schools started to reopen. In total, 1,848 families took part in the survey. For the present paper we included data from 1,812 families who responded in wave 9 and for whom data from waves 7 and 8 were available (regardless of responding parent). In that sample, we treated values from waves 8 or 9 as missing if the responding person changed in the corresponding waves.

We applied a weighting procedure to account for a sample bias toward higher-educated parents and misbalances in other sample characteristics to increase representativeness of the results (for details, see also section ‘‘Analytic strategy’’). In wave 9, the mother was the responding parent in 98% of the families; the mean age of 39 years (weighted; 40 years unweighted)^1^. The responding parent had approximately 13.73 years of education (*SD* = 2.32; unweighted: mean = 15.43, *SD* = 2.29), and around 22% (weighted; 16% unweighted) of the responding parents had a migration background (born outside of Germany). Around 55% (weighted; 50% unweighted) of the participating children were female in wave 9.

### Research Instruments

#### Dependent Variables

##### Self-Assessment of Supporting Abilities

In the COVID-19 questionnaire in wave 9, the responding parents were asked to assess their own ability to support their child’s learning at home at this time during school closure in terms of learning content: “How do you assess your ability to support your child with content during the school closure to help your child learn at home at this time?”. They could respond using a 4-point-scale from “completely sufficient” to “completely insufficient” (the scale was reversed for all analyses).

##### Perceived Stress

The second dependent variable was the perceived stress of the responding parent. The parents were asked to answer the following statement on a 5-point-scale, ranging from “does not apply at all” to “does completely apply”: “I was very stressed by the school closure and the demands of homeschooling.”

#### Predictors

##### Structural Aspects

As structural aspects the following two variables were considered:

The first variable was the years spent in education by the responding parent (as a function of CASMIN; König et al., 1988).

Second, the highest International Socioeconomic Index of Occupational Status (ISEI-08, Ganzeboom et al., 1992) of both parents was included (HISEI). The ISEI hierarchizes the occupational status of a person’s last occupation according to the average earning and education of individuals with the respective occupation. The HISEI was utilized as an indicator of the SES of the family.

##### Process Aspect

The following process aspect was used as indicator of parental investment in the child’s education:

Facet of the HLE. Based on the FIM, we included joint stimulating activities to cover former facets of the HLE. Joint activities were assessed in wave 8 (first grade) using four items (Melhuish et al., 2008). For example, the parents were asked how often they (or someone else in the household) read or tell stories to the child (see Supplementary Table 1 for all items as well as the indices of the measurement model). The responses on the 8-point-likert scale ranged from several times a day to never (the scale was reversed for all analyses). Although the internal consistence (Cronbach’s alpha) was questionable (0.62), in confirmatory factor analysis (CFA) the measurement model showed an acceptable fit (for the utilized cutoff criteria and detailed information see section “Analytic strategy”).

##### Psychological Characteristics of the Parents

###### Psychological Stress

Psychological stress was measured in wave 7, last year before the children started school. The psychological stress measure included five items. First, the parents were asked to specify how often they had felt depressed or sad in the last 4 weeks using a 5-point Likert scale with responses ranging from “never” to “always” (adaption of SF-12 from SOEP-Study; Ware et al., 2002; Goebel et al., 2019). Second, aspects of parenting strain were includes, such as if the parents suffered from being restricted to role as mother/father. Here, the 4-point Likert scale ranged from “completely disagree” to “completely agree” (adaption from SOEP-Study; Goebel et al., 2019; for negative items the scale was reversed for all analyses). Cronbach’s alpha was 0.68; the measurement model again showed an acceptable fit. No data were collected on psychological stress in wave 8. Hence, psychological stress and the structural aspects were measured at the same time point.

##### Psychological Characteristics of the Child

School-related independence of the child. As an important characteristic of child, the school-related independence of the child was used (adaption of FEESS from BiKS-Study; Rauer and Schuck, 2003; von Maurice et al., 2007). In wave 8, parents rated items such as “the child does most of his/her homework on his/her own” using a 4-point Likert scale ranging from “does not apply at all” to “does completely apply” (the scale was reversed for negative items for all analyses). Overall, the construct was measured using three items; Cronbach’s alpha was 0.67. Results of CFA were uninformative for the measurement model due to the low number of indicators and resulting zero degrees of freedom (West et al., 2012).

###### Self-Regulation

Self-regulation of the child was measured in wave 7 using three items. To this end, parents rated aspects such as whether the child calms down relatively quickly if it doesn’t get what he or she wants, using a 4-point Likert scale ranging from “does not apply” to “does apply” (the scale was reversed for negative items for all analyses; German version of the California-Child-Q-Sort, adaption from BiKS-Study; Göttert and Asendorpf, 1989; von Maurice et al., 2007). Cronbach’s alpha was 0.68. As with school-related independence of the child, the number of indicators of the measurement model was too low to obtain meaningful model fit indicators in CFA.

#### Control and Additional Variables

To take demographic factors and the contextual frame of parental experiences during the pandemic into account, we included a number of control variables. First, the sex and age of the responding parent as well as the sex of the child were included. Further, to control for different household settings, we collected information about the number of children under 14 years in the household in waves 8 and 9.

The extent to which a child struggled with homeschooling was assumed to be a crucial covariate of the outcomes. If it is also a mediator of structural, processual, and personal effects on the outcomes, could not be tested in the present study. As data on the child’s struggles with homeschooling were collected at the same time point as the parent’s report on their ability to provide support with homeschooling and deal with stress, we can only look at the association between these factors and cannot claim a specific directionality of the effects. Due to missing information about employment status during the lockdown (for relevance, see Porsch and Porsch, 2020) for the whole sample, we considered the employment situation of the responding parent before lockdown at wave 9. We also considered the childcare situation during lockdown and the perceived control of the respondent over his or her live during the pandemic.

In addition to the structural predictors discussed above, another structural aspect of the family that was found to correlate with COVID-19- related stressors such as parental stress and bad health, is being part of a minority (Brown et al., 2020). As the investigated minorities (e.g., African and Latin Americans) were also socioeconomically disadvantaged, we assumed the main effect to be attributed to these structural inequalities. Therefore, belonging to a minority was not the focus of our research. Nevertheless, we included migration background in additional analyses to check for independent effects of being part of a minority (the results of the models with weighted and unweighted data can be found in the Supplementary Figures 2, 3). Migration background was operationalized as the respondent being born outside of Germany.

### Analytic Strategy

Already in wave 7, the Starting Cohort 1 of the NEPS showed a severe bias toward higher-educated respondents (approximately 47% of responding parents hold an academic degree) due to a higher rate of attrition among lower-educated parents in the panel. To limit bias and increase representativeness, we applied a weighting procedure (see Würbach et al., 2021) to account for disproportionalities between our sample in wave 9 and the micro-census quota (Lüttinger and Riede, 1997). The sample was adjusted to comply with the current micro-census quota in terms of education (ISCED; UNESCO Institute for Statistics, 2012), country and year of birth of the parent, federal state, size of the place of residence and employment. After an iterative weighting procedure, each case received an individual weighting factor. In this paper, sample characteristics are presented for both the unweighted and weighted data. Statistical analyses were conducted with the weighted and the unweighted data set. The results of the analysis with weighted data are presented in the paper; results of the analysis with unweighted data are presented in the Supplementary Material.

**FIGURE 1:**
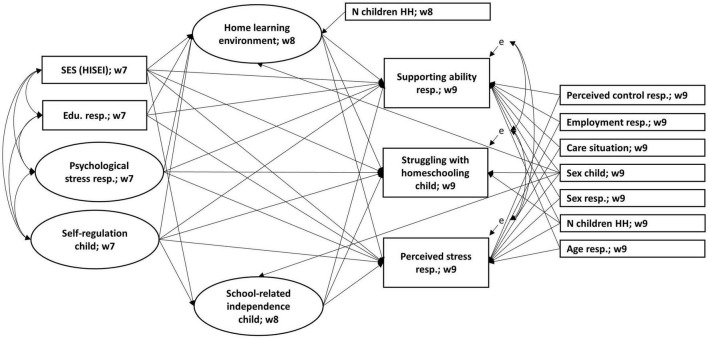
Analytic model. All assumed and estimated paths are shown.

For the evaluating of the measurement models of the latent variables we performed CFA and utilized the cutoff criteria proposed in Hu and Bentler (1999; CFI ≥ 0.95, TLI ≥ 0.95, RMSEA ≤ 0.06) to identify acceptable model fit (the model fit indicators of all measurement models can be found in the Supplementary Table 1). We applied structural equation modeling (SEM) to investigate the effect of the structural aspects of the family measured at wave 7 on parental self-assessed ability to support the child and the perceived stress of the parent during the pandemic in wave 9. The process characteristics of the family and psychological characteristics of the responding parent and the child measured in wave 8 (HLE, school-related independence of the child), were modeled as mediators of the structural effects on parental outcomes. The process and psychological characteristics assessed in wave 7 were treated as independent variables (psychological stress of the parent, self-regulation of the child). As control variables, we considered the parent’s sex and age, the child’s sex, number of children under 14 years in the household, employment situation of the responding parent before lockdown, childcare situation during lockdown, the perceived control of the responding parents, and the child’s struggles with homeschooling. The analytic model is shown in Figure 1.

Further, we covaried the residuals of the two dependent variables, as there could be a reciprocal effect not explained by the common predictors and controls. Due to a lack of clear expectations regarding the directionality of influences because the measurement was carried out at the same time point, we covaried the residuals of the child’s struggles with homeschooling with the residuals of both dependent variables. Similar to for the dependent variables, we predicted the child’s struggles with homeschooling with the preceding structural, processual, and personal factors in order to take into account possible pathways of moderation.

For all analyses, we modeled predictors as latent variables where possible (except years of education and SES of the family). All controls were included as manifest variables.

Structural equation modeling were calculated using Stata 15.1. Missing values were treated with Full Information Maximum Likelihood Estimation (FIML). Postestimation procedures for SEM such as CFI, TLI, and RMSEA are inappropriate for survey estimation results using weighted data, as there is no sample likelihood value (StataCorp, 2021, p. 113). Hence, to obtain model fit indicators, we run our main model again with non-weighted data.

## Results

### Descriptive Analyses

Table 1 shows the descriptive characteristics of the analyzed variables. Constructs measured with multiple indicators were represented by a mean index (find all indicators in the Supplementary Material 1). The mean values of self-assessed supporting abilities and perceived stress in wave 9 show a high self-efficacy expectation in parents with regard to their ability to support their child. However, perceived stress was also situated in the upper area of the scale.

**TABLE 1:** Descriptive statistics.

	*N*	Minimum	Maximum	*M*	*SD*
***Dependent variables***					
Self-assessed supporting abilities; w9	1,739	1	4	3.65	0.61
Perceived stress; w9	1,743	1	5	3.42	1.33
***Structural aspects***					
Education of respondent, years (CASMIN); w7	1,717	9	18	13.73	2.32
SES (highest ISEI of family); w7	1,810	14	89	55.38	19.81
***Process aspect***					
Home learning environment (mean of indicators); w8	1,708	1	7.75	4.78	1.23
***Characteristics of the parent***					
Frequency feeling depressed; w7	1,725	1	5	2.24	0.98
Parenting strain (mean of indicators); w7	1,725	1	3.5	1.60	0.46
***Characteristics of the child***					
Self-regulation (mean of indicators); w7	1,637	1	4	2.95	0.64
School-related independence (mean of indicators); w8	1,658	1	4	3.21	0.59
***Control variables***					
Child struggling with homeschooling; w9	1,742	1	5	2.01	1.02
Number of children under 14 years in household; w9	1,746	1	7	2.01	1.03
Number of children under 14 years in household; w8	1,707	1	7	2.01	0.97
Perceived control (1 = high, 5 = low); w9	1,745	1	5	3.48	1.22
Age respondent (years); w9	1,773	26	57	39.15	5.62
Sex respondent (1 = male); w9	1,748	1	2	1.98	0.13
Sex child (1 = male); w9	1,804	1	2	1.55	0.50
Care situation during lockdown (0 = only others caring; 1 = me and others caring); w9	1,748	0	1	0.84	0.36
Employment before lockdown (0 = not or spare-time employed; 1 = part- or fulltime employed); w9	1,746	0	1	0.83	0.38

*Indicators collected in wave 7 (w7), wave 8 (w8), and wave 9 (w9). Mean and SD estimated with weighted data.*

Table 2 presents the correlations between the manifest predictors and outcomes and the mean indices of the multiple indicator constructs. Interestingly, the two dependent variables, self-assessed supporting abilities and perceived stress, showed no significant correlation (*r_*s*_* = –0.03, *p* > 0.10). Both structural characteristics correlated with the ability of the responding parent to provide support. That means that parents with a higher education and socioeconomic background evaluated their abilities to support their child’s learning during the school closures higher than parents with lower education and socioeconomic background. Of other assumed predictors, only psychological stress and the previously assessed self-regulation of the child showed an association with the supporting abilities (the child’s previous self-regulation surprisingly showed a negative association). Perceived stress correlated with the SES of the family as well as three of the other predictors. The strongest association was with previous psychological stress. Only joint activities, as a facet of a stimulating HLE prior to the pandemic, did not correlate with the perceived stress of the respondent. All correlations including control variables can be found in the Supplementary Table 2.

**TABLE 2:** Correlations (Spearman rho).

	2	3	4	5	6	7	8
1 Supporting abilities (w9)	−0.03	0.31***	0.21***	0.04	−0.11***	−0.06*	0.01
2 Perceived stress (w9)	1	0.05^+^	−0.13***	−0.03	0.20***	−0.06*	−0.16***
3 Education (w7)		1	0.53***	0.04	0	−0.10***	0.15***
4 SES (w7)			1	0.06*	0.08**	−0.19***	0.22***
5 HLE (w8)^1^				1	−0.11***	−0.02	−0.06*
6 Psychological stress (w7)^2^					1	−0.22***	−0.09**
7 Self-regulation (w7)^1^						1	0.15***
8 School-related independence (w8)^1^							1

SES, socioeconomic status, highest ISEI of family; HLE, home learning environment; w7, wave 7; w8, wave 8; w9, wave 9. ^1^Mean of indicators. ^2^Mean of standardized indicators. Correlations estimated with weighted data. ^+^p < 0.10, *p < 0.05, **p < 0.01, ***p < 0.001.

### Structural Equation Modeling of Dealing With the Pandemic

The main model, considered as a more complex model than a CFA (Weiber and Mühlhaus, 2010; West et al., 2012), demonstrated an acceptable fit with the unweighted data^2^ [Chi^2^ (248) = 643.76, CFI = 0.93, TLI = 0.91, RMSEA = 0.03]. All estimates of the structural model (performed with weighted and unweighted data) are presented in the Supplementary Table 3. Due to the bias in the sample in favor of higher-educated parents, the effects of the model calculated with unweighted data should be interpreted with caution.

**FIGURE 2:**
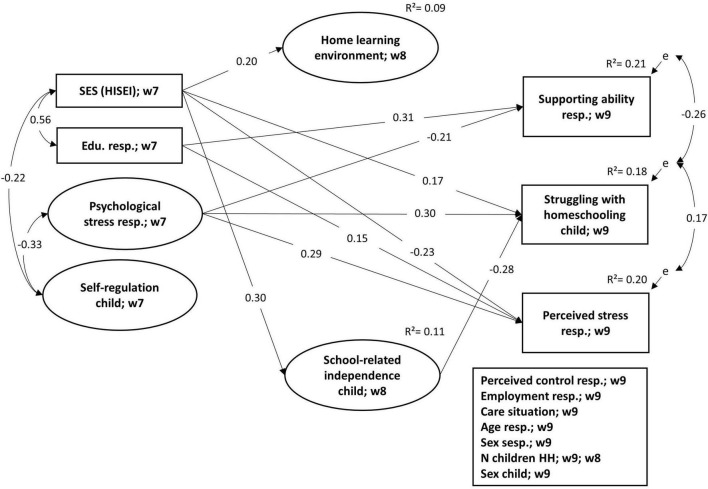
Results of the structural equation model for self-assessed supporting ability and perceived stress during the school closure (algorithm mlmv). Only the significant standardized coefficients are presented (*p* < 0.05). SES, socioeconomic status, highest ISEI of the family. Edu. resp., education of the respondent in years. w, wave. Estimated with weighted data. Measurement models of latent variables in the Supplementary Table 1. Model fit of estimation with unweighted data: *N* = 1,812; Chi^2^ (248) = 643.76, CFI = 0.93, TLI = 0.91, RMSEA = 0.03.

Figure 2 shows all significant standardized estimates of the main model based on the weighted data (for the model with unweighted data see Supplementary Figure 1). We found a significant effect of the respondent’s education on his or her ability to provide support (0.31); hence better-educated parents evaluated their ability to support the learning of their child higher than lower-educated parents. On the other hand, the second structural characteristic, the SES of the family, showed no effect. Most of the process and psychological characteristics did not predict self-assessed supporting abilities. Only the preceding psychological stress of the respondent showed a negative effect (–0.21); hence respondents who reported more psychological stress before the pandemic evaluated their ability to provide support during the pandemic lower than respondents with lower psychological stress prior to the pandemic. Further, a residual covariance of –0.26 was found with the child’s struggles to deal with the school closure. In other words, parents reported a lower ability to provide support if their child struggled more with the situation, even after controlling for structural, processual, and psychological determinants and control variables. Further, none of the other control variables – namely age and sex of the respondent, sex of the child, number of children under 14 in the household, employment before lockdown, childcare situation and perceived control – showed a significant association with ability to support. No significant association was found between supporting abilities and perceived stress. Altogether, the considered constructs explained 21% of the variation of the respondent’s ability to provide support.

The second dependent variable – perceived stress – was predicted negatively by the SES of the family (–0.23) and positively by the years of education (0.15). Thus, in families with a higher SES, the responding parent perceived lower stress. On the other hand, better-educated respondents reported more perceived stress during the pandemic than lower-educated parents. In line with our assumptions, respondents reporting more psychological stress before the pandemic also reported more stress during the pandemic (0.29). Further, the perceived stress of the respondent not explained by the predictors and controls (residuals) covaried with the residuals of the child struggling during the pandemic (0.17). Consequently, parents, who reported more perceived stress also described their child as struggling more with the pandemic situation. Again, no significant associations between the other control variables and perceived stress were found. Taken together, the considered predictors explained 20% of the variance of perceived stress.

Further, the SES of the family also predicted the child’s struggles (0.17), facets of the HLE (0.20), and school-related independence (0.30). Besides the remarkable weaker positive effect of the family’s SES, the child’s struggles during the pandemic were also predicted by the parent’s previous psychological stress (0.30) and school-related independence of the child (–0.28). Thus, school-related independence mediated a negative effect of the family’s SES on the child struggling during the lockdown (indirect effect: –0.08). The other control variables showed no significant association to the child struggling. Overall, 18% of the variance of the child’s struggles with the homeschooling situation was explained by the considered variables. In addition, 9% of the variance in home learning environment facets (here, a significant effect of child sex was found in favor for the girls) as well as 11% of school-related independence were explained by the independent variables and controls.

## Discussion

The COVID-19 pandemic has posed many challenges, especially for families. Already early in the pandemic, the public and scientific community started voicing concerns about the consequences of the pandemic on children and their development. However, parents have also been affected by the pandemic, which in turn influences their children (Conger et al., 1992; Bronfenbrenner and Morris, 2006; Conger and Donnellan, 2007; Kluczniok et al., 2013; Prime et al., 2020). The conceptual framework representing how the pandemic may affect children, parents, and families assumes not only a mutual influence between children and parents, but also a role of pre-existing vulnerabilities in how parents and children cope with the pandemic (Prime et al., 2020). Against this background, the present paper investigates the effects of the structural, processual, and psychological characteristics of parents and children on how parents of elementary students dealt in the pandemic longitudinally.

Based on the FSM (Conger et al., 1992) and the FIM (Conger and Donnellan, 2007), we expected the socioeconomic background of families and the education of the main caregiver to predict the responding parent’s perceived stress and self-assessed ability to support his or her child during school closure in spring 2020. In line with the theoretical models, we further assumed that previous parental psychological stress, the child’s psychological characteristics, and previous parental investment in stimulating activities would have an effect on perceived stress and the parent’s ability to provide support.

### The Role of Structural Characteristics in Handling the Challenges of the School Closures During the Pandemic

For our first research question, which addressed the role of structural characteristics, we hypothesized that SES would predict perceived stress and that the responding parent’s education would predict his or her ability to provide support. The results of the structural equation model confirmed a significant negative effect of SES on parents’ perceived stress during the pandemic. This is in line with the theoretical models and implies unobserved mediating effects playing into this finding in the form of economically determined living conditions (e.g., overcrowding – especially in homeschooling situations-, problems making ends meet and having to cut back on necessary expenses; see, e.g., Simons et al., 2016). As the NEPS does not provide such information, further research is needed to investigate the effects of living conditions and possible mediating effects. We did not find an effect of the family’s SES on the responding parent’s self-assessed ability to support the child. Surprisingly, the family’s SES increased the extent to which the child struggled with homeschooling significantly, which in turn was associated with both parental outcomes in the pandemic. This finding is hard to classify. Higher expectations toward the child’s academic performance and behavior as well as stronger monitoring of the child’s learning in higher SES families (see, e.g., Stull, 2013; Guo et al., 2018) can be discussed as potential explanations. Nonetheless, a negative indirect path from family’s SES on the child’s struggles with homeschooling was also identified, mediated by the child’s school-related independence. As data on the child’s struggles were collected at the same time point as the parental outcomes, a mediation of the SES effects on parental perceived stress and ability to provide support by the child’s struggles with homeschooling can only be taken into consideration. Due to the pandemic, there was another lockdown in winter 2021 in Germany. Hence, possible mediating effects can be verified by further research with a longitudinal view on the pandemic.

Furthermore, and contrary to our hypothesis, the responding parent’s education was found to have a weaker yet also significant positive effect on perceived stress. This finding is surprising, but has also been reported in other studies (see Porsch and Porsch, 2020). In contrast, Zinn and Bayer (2021) reported the effect the other way around but did not control for SES of the family. It is important to keep in mind that this effect was only found when we controlled for the SES of the family in the SEM. In the bivariate correlations, the association between the parent’s education and perceived stress did not reach significance. A possible explanation could be that higher-educated parents have a higher personal expectation to deal with the pandemic situation, leading to increased stress (Parker and Mills, 1996; Smith et al., 2021).

Based on empirical evidence (Bol, 2020; Sari et al., 2021), we expected the education of the responding parent to be the strongest structural predictor of self-assessed ability to provide support. The results of the structural equation model confirmed this expectation. Better-educated parents reported a higher ability to support the learning of their child than lower-educated parents. As already discussed by Zinn and Bayer (2021), the reason could be that higher-educated parents are in general more familiar with the educational system.

### The Role of the Home Learning Environment in Handling the Challenges of the School Closures During the Pandemic

Based on the FIM (Conger and Donnellan, 2007), our second research question focused on the role of investment in the HLE prior to the pandemic as a predictor of the ability to provide support during the lockdown. Contrary to our hypothesis, we found no effects of the HLE, which was measured as the frequency of carrying out stimulating activities with the child one year prior to the lockdown, on the self-assessed ability to provide support during the first wave of the pandemic. A possible explanation for the lack of association are the differences in content between the operationalization of these two kinds of parental investment in the child’s education. This result can give a hint that facets of parental investment other than stimulating activities played a role during the first school closure (e.g., information about and involvement with schools’ learning support and material). It’s up to further research to set a clearer focus on different facets of the HLE and their role for dealing with the challenges caused by the pandemic.

Consequently, our expectation that the HLE would partially mediate the structural effects of parent’s education and socioeconomic background on the ability to provide support has to be rejected. Nevertheless, we identified a positive significant effect of the family’s SES on the HLE. It is not surprising that families with a higher SES report more frequent stimulating activities, as social differences in aspects of the HLE have often been found (Lehrl et al., 2012; Hayes and Berthelsen, 2020) and are already evident in the first years of life (Attig and Weinert, 2020).

### The Role of Psychological Stress in Handling the Challenges of the School Closures During the Pandemic

Our third research question investigated whether a parent’s psychological stress prior to the pandemic acts as a predictor of perceived stress during school closure. We found a clear positive effect of psychological stress measured 2 years before the pandemic on perceived stress during school closure. This finding replicates a number of previous empirical studies investigating the antecedents of parental mental health during the pandemic with cross-sectional data (Brown et al., 2020; Davis et al., 2020; Chartier et al., 2021). Hence, the present findings clearly confirm the association between previous psychological stress and perceived stress during the pandemic longitudinally (for roughly similar longitudinal findings, see Racine et al., 2021).

Beyond that, a significant effect of previous psychological stress on ability to provide support during the pandemic was detected. This kind of crossover influence is not formalized in the Family Stress or the FIM. For the FSM it could be argued that supporting the child is parent–child interaction and therefore is expected to be directly and indirectly influenced by parents’ stress (Conger et al., 1992). The self-assessed supporting ability as a self-efficacy belief, however, is rather a possible predictor of actual interaction. Nevertheless, the association between parental stress and self-reported ability to support is identified in empirical studies (e.g., Kohl et al., 2000; Baranov et al., 2020). In a cross-sectional study conducted in Germany, Porsch and Porsch (2020) found that parental self-efficacy beliefs on their ability to provide support with the child’s schoolwork during the pandemic was a negative predictor of parental mental stress. As they had only data from the time of the pandemic, a causal interpretation of these results was not possible. Our longitudinal data point to previous mental stress rather being a predictor of self-assessed ability to provide support, than the other way round. Further research is required to confirm this finding.

Another possible pathway of the effects of previous psychological stress on both parental outcomes is the noticeable increasing effect of psychological stress on the child’s struggles with the homeschooling situation, which is associated with perceived stress and the ability to provide support during the pandemic. However the question arises as to whether this effect is due to a biased evaluation of the child’s behavior by mentally strained parents (see, e.g., Najman et al., 2001).

Based on the theoretical models (Conger et al., 1992) and empirical evidences (Brown et al., 2020; Chartier et al., 2021), it can be assumed that previous psychological stress is a mediating factor of the structural effects on the perceived stress during the lockdown. However, as previous psychological stress was measured on the same occasion as the structural background factors, no mediation could be tested. Furthermore, the covariance between SES as well as the responding parent’s education and psychological stress did not attain significance; therefore, the data did not support any further plausible assumptions of a mediation.

### The Role of Characteristics of the Child in Parents’ Handling of the Challenges of the School Closures During the Pandemic

Our last question focused on whether the characteristics of the child predicted parental handling of the challenges of school closure. In accordance with Abidin’s (1992) Parenting Stress Model, we expected the child’s social skills and problematic behavior to predict parental outcomes in the pandemic. The child’s self-regulation and school-related independence both showed no significant influence on parents’ self-assessed ability to support the child with schoolwork or perceived stress during school closure. Nevertheless, the child’s previous school-related independence decreased the extent to which the child struggled with the homeschooling situation, which in turn was associated with both parental outcomes. A possible mediating effect can be discussed.

To sum up, the present study utilizes pre-pandemic measurements of families’ structural and process characteristics and the psychological characteristics of the parents and the child to explain the perception of parental challenges during the COVID-19 pandemic. It thus provides a longitudinal perspective on the antecedents of parents’ ability to handle these challenges. In addition, it includes the central mediators from two different prominent models explaining the mechanisms of the impact of structural background on child development (Conger et al., 1992; Conger and Donnellan, 2007), thus providing a benefit by focusing on several pathways of mediation. Furthermore, the study offers opportunities to investigate interdependencies between parental investment and parental stress – in terms of both cross-sectional and longitudinal associations. Adding to previous research, the results of the present paper show that pre-existing social differences as well as pre-existing psychological stress influence how parents deal with the pandemic. Nevertheless, further research is needed to analyze how these factors influence child development, particularly with regard to the still ongoing pandemic accompanied with the challenges for families.

As the Newborn Cohort Study of the NEPS collected further data on the COVID-19 pandemic in wave 10 after the second school closure in winter 2020/2021, future research can use the potential of longitudinal modeling to investigate the development of parental perception of challenges during the pandemic as well as its predictors and moderators. The cognitive and socio-emotional outcomes of the child could be included in future studies. This might potentially provide empirical backup for the theoretical assumptions that parental investment and stress in the pandemic predict the child’s development and mediate structural effects of the family (Conger et al., 1992; Conger and Donnellan, 2007). In addition, information is available on the schools’ support of home learning which could be included in future research.

### Strength and Limitations

Utilizing data of the Newborn Cohort of the NEPS, this study contributes to research on the challenges posed on parents of second-graders by the COVID-19 pandemic by using longitudinal data from a large scale survey. Drawing on the benefits of structural equation modeling, the present study investigates how parents dealt with the cognitive and emotional challenges of the pandemic in a joint model. Further, as the NEPS provides longitudinal data, not only information assessed during the pandemic was considered, but also data from the years before the pandemic. This enabled us to analyze the impact of pre-existing social differences in the families as well as the process characteristics of the family and psychological characteristics of parent and child. Another strength of this study is the use of weighting procedures in the main model before interpreting the effects, which limited bias and increased representativeness of results obtained with the present sample under study. Further, we applied FIML to deal with missing values which are inevitable in longitudinal data.

However, the present study is not without limitations. First of all, our dependent constructs were operationalized by only one variable each. This means that our items only represent a narrow aspect of the respective constructs under investigation. Measurements with one variable are more vulnerable to measurement errors as well as to unknown biases in meaning and interpretation than measurements with more variables. Furthermore, single item indicators suffer from a low sensitivity. This means that a single item provides fewer points of discrimination than multiple items (Nunnally, 1978; McIver and Carmines, 1981). Hence, with regard to that, our results must be interpreted with caution. For example, it could be assumed that the missing association between preceding investments in the HLE and the ability to support during the pandemic is a consequence of this reduction of facets of the construct. Unfortunately, for economic reasons, the amount of survey time was limited, so only a few questions could be added to assess how parents were dealing with the pandemic.

In addition, even if the study follows a longitudinal perspective, we did not utilize strictly longitudinal methods. As the constructs were measured using different operationalizations over different waves, we could not apply repeated measurement methods and report mean level differences and trajectories.

A third limitation was the fact that the mother was the respondent of the survey in most cases. Hence, the sample contains only limited information about how fathers dealt with the situation. As already mentioned, in the first wave, the mothers were asked to be the respondent, and in most cases the mothers remained the respondent. Consequently, this sample does not allow research on sex differences in dealing with the pandemic, but that was not the focus of our paper. Nevertheless, the present study loses variation due to this specific sample, and the results should be interpreted with caution. Future research should take into account differences due to the sex of the respondent.

## Conclusion

Taking family processes as well as personal and child characteristics into account, the present paper analyzed social differences in how parents of second-graders dealt with the challenges of the pandemic. To this end, it focused on two aspects, namely parents’ perceived stress and their ability to support the learning of their child. As our analyses are based on longitudinal data, our results shed some light on the directionality of effects between structural, processual and psychological family conditions and parents’ experience of the homeschooling situation. Besides the corroboration of results pointing out the crucial role of family’s SES, our study shows some surprising results. Though the association between parental stress and parents’ ability to provide support was found before (e.g., Kohl et al., 2000; Baranov et al., 2020; Porsch and Porsch, 2020), the relationship was not examined in the context of the COVID-19 pandemic or it was assumed that supporting ability influences mental stress (Porsch and Porsch, 2020). Our results showed that the preceding psychological stress of the parents predicted the supporting abilities during the school closures. Furthermore, parental education could not be confirmed as a protective factor against mental stress once the occupational status was controlled for, but on the contrary, higher education seems to increase parental perceived stress. This holds theoretical as well as practical implications.

For research on families’ experiences in the COVID-19 pandemic, this suggests to regard parental stress as a predictor when investigating parental support of children’s learning, even when following explanatory models not explicitly considering the role of mental health. Secondly, taking different aspects of SES into account can be recommended. Different facets of SES, like the occupation determining the family’s material resources and education representing the cultural aspect, could be shown to have differential effects on the same outcomes in the pandemic.

Addressing the practical provision of support for families challenged in the pandemic, this draws attention on the mental health status of families. Family support after the school closures should not only focus on catching up on children’s learning gaps in socially disadvantaged families. Providers should also take into account the potential emotional problems of parents and in the family system as a whole that could have arisen in the pandemic and may in turn influence the child’s learning. For example, providers of support and educators could encourage and help building social support networks between struggling families and connect them with community support resources like childcare offers or counseling.

## Data Availability Statement

This article analyzed the data from the National Educational Panel Study in Germany. The anonymized data are available for the scientific community at: https://www.neps-data.de.

## Ethics Statement

The NEPS study is conducted under the supervision of the German Federal Commissioner for Data Protection and Freedom of Information (BfDI) and in coordination with the German Standing Conference of the Ministers of Education and Cultural Affairs (KMK) and—in the case of surveys at schools—the Educational Ministries of the respective Federal States. All data collection procedures, instruments, and documents were checked by the data protection unit of the Leibniz Institute for Educational Trajectories (LIfBi). The necessary steps are taken to protect participants’ confidentiality according to the national and international regulations of data security. Participation in the NEPS study is voluntary and based on the informed consent of participants. This consent to participate in the NEPS study can be revoked at any time. All parents of the Newborn Cohort of the NEPS give their agreement for participation and answering questions during the assessments as well as written consent for participating in the video-taped measures to each measurement point.

## Author Contributions

MV and MA contributed to the conception and the design of the manuscript. MV performed the statistical analysis. Both authors contributed to the article and approved the submitted version.

## Conflict of Interest

The authors declare that the research was conducted in the absence of any commercial or financial relationships that could be construed as a potential conflict of interest.

## Publisher’s Note

All claims expressed in this article are solely those of the authors and do not necessarily represent those of their affiliated organizations, or those of the publisher, the editors and the reviewers. Any product that may be evaluated in this article, or claim that may be made by its manufacturer, is not guaranteed or endorsed by the publisher.

## Footnotes

1 Due to questions about the pregnancy and breastfeeding, the mother was aimed to be the respondent in the first wave. After that, a change of respondent was possible only in few exceptions. This explains why in the most cases the mother is still the respondent in the Newborn Cohort of the NEPS.

2 Due to technical reasons, the common model fit indicators could not be calculated with the weighted data (StataCorp, 2021, p. 113). For that, we included at least the model fit of the estimation with unweighted data.
