# Supplementary material for: Carrying the Burden Into the Pandemic – Effects of Social Disparities on Elementary Students’ Parents’ Perception of Supporting Abilities and Emotional Stress During the COVID-19 Lockdown
Source: Front Psychol. 2022 Jan 11;12:750605. doi: 10.3389/fpsyg.2021.750605 (PMC8787329; doi:10.3389/fpsyg.2021.750605)
Supplement: Supplementary file 1 [file Data_Sheet_1.docx]

Supplementary Material

# Latent predictors and their measurement models

*Supplementary Table 1.* Indicators and measurement models.

| Constructs | Num-ber of items | Item details | Response scale | Cron-bachs alpha | Coefficient (std)/ SE | | Model Fit (unwghtd) | | | | |
| --- | --- | --- | --- | --- | --- | --- | --- | --- | --- | --- | --- |
|  |  |  |  |  | wghtd | unwght | N | Chi² (df) | RMSEA | CFI | TLI |
| ***Process aspect*** |  |  |  |  |  |  |  |  |  |  |  |
| Home learning environment (HLE) | 4 | You or someone else read(s) to <the child> at home. | From 1 “never” to 8 “several times a day” | 0.62 | 0.39/ 0.08 | 0.39/ 0.03 | 1,708 | 9.85 (2) | 0.048 | 0.988 | 0.964 |
|  |  | You or someone else tells <the child> stories at home, such as fairy tales you have made up or retell. |  |  | 0.53/ 0.14 | 0.48/ 0.03 |  |  |  |  |  |
|  |  | You or someone and <the child> look at books about nature together at home, for example, about animals, experiments, plants, or technology. |  |  | 0.81/ 0.13 | 0.74/ 0.03 |  |  |  |  |  |
|  |  | You or someone else and <the child> identify or name animals or plants together. |  |  | 0.45/ 0.10 | 0.47/ 0.03 |  |  |  |  |  |
| ***Characteristics of the Parents*** |  |  |  |  |  |  |  |  |  |  |  |
| Psychological stress | 5 | How often in the last 4 weeks did you feel depressed or sad? (standardized)^1^ | From 1 „never“ to 5 „always” | 0.68 | 0.45/ 0.08 | 0.50/ 0.03 | 1,725 | 8.14 (3) | 0.032 | 0.996 | 0.987 |
|  |  | I am very satisfied with my role as a mother/father. (reversed, standardized)^2^ | From 1 "completely disagree” to 4 “completely agree” |  | 0.53/ 0.08 | 0.32/ 0.03 |  |  |  |  |  |
|  |  | I often feel I have not strength or energy. (standardized)^1^ | From 1 "completely disagree” to 4 “completely agree” |  | 0.58/ 0.08 | 0.52/ 0.03 |  |  |  |  |  |
|  |  | I am suffering from being restricted to my role as a mother/father. (standardized)^2^ | From 1 "completely disagree” to 4 “completely agree” |  | 0.47/ 0.08 | 0.51/ 0.03 |  |  |  |  |  |
|  |  | I often feel alone. (standardized) | From 1 "completely disagree” to 4 “completely agree” |  | 0.64/ 0.08 | 0.71/ 0.03 |  |  |  |  |  |
| ***Characteristics of the Child*** |  |  |  |  |  |  |  |  |  |  |  |
| School-related independence of the child | 3 | Now I would like to ask you some questions about <the child>'s everyday school life […].  <The child> does most of his homework on his own. | From 1 “does not apply at all” to 4 “does completely apply” | 0.67 | 0.72/ 0.14 | 0.77/ 0.02 | 1,658 | 0(0) | 0 | 1 | 1 |
|  |  | <The child> needs a lot of help with the homework. (reversed) |  |  | 0.77/ 0.13 | 0.87/ 0.02 |  |  |  |  |  |
|  |  | For <the child>, many tasks at school come easily. |  |  | 0.47/ 0.13 | 0.53/ 0.02 |  |  |  |  |  |
| Self-regulation | 3 | My child often reacts excessively angrily to minor disappointments. (reversed) | From 1 “does not apply” to 4 “does apply” | 0.68 | 0.73/ 0.09 | 0.71/ 0.02 | 1,637 | 0 (0) | 0 | 1 | 1 |
|  |  | My child gets angry easily. (reversed) |  |  | 0.79/ 0.15 | 0.86/ 0.03 |  |  |  |  |  |
|  |  | My child calms down relatively quickly if it doesn't get what he/she wants. |  |  | 0.41/ 0.08 | 0.44/ 0.03 |  |  |  |  |  |

Note. All indicators standardized for CFA.^1/2^ Due to model optimization errors of indicators covaried for CFA.

# Additional descriptive analyses

*Supplementary Table 2.* Correlations (Spearman Rho).

|  |  | 2 | 3 | 4 | 5 | 6 | 7 | 8 | 9 | 10 | 11 | 12 | 13 | 14 | 15 | 16 | 17 |
| --- | --- | --- | --- | --- | --- | --- | --- | --- | --- | --- | --- | --- | --- | --- | --- | --- | --- |
| 1. | Supporting abilities (w9) | -.03 | .31*** | .21*** | .04 | -.11*** | -.06* | .01 | -.21*** | -.15*** | -.13*** | .02 | .11*** | .17*** | -.08** | -.03 | .16*** |
| 2. | Perceived stress (w9) | 1 | .05^+^ | -.13*** | -.03 | .20*** | -.06* | -.16*** | .20*** | -.13*** | -.10*** | .03 | .07** | .09*** | .07** | .13*** | .11*** |
| 3. | Education in years (w7) |  | 1 | .53*** | .04 | 0 | -.10*** | .15*** | .03 | .09** | .04 | .02 | .14*** | .12*** | .04 | .09*** | .30*** |
| 4. | SES (w7) |  |  | 1 | .06* | .08** | -.19*** | .22*** | .09*** | -.04 | .01 | .02 | .06* | .08** | .07** | -.04 | .22*** |
| 5. | HLE (w8)^1^ |  |  |  | 1 | -.11*** | -.02 | -.06* | .04 | .04 | -.02 | .05* | -.07** | -.06* | -.18*** | .22*** | .054 |
| 6. | Psychological stress (w7)^2^ |  |  |  |  | 1 | -.22*** | -.09** | .29*** | -.07** | -.003 | .18*** | -.11*** | .14*** | .01 | .07** | -.03 |
| 7.. | Self-regulation (w7)^1^ |  |  |  |  |  | 1 | .15*** | -.19*** | .02 | .05+ | -.02 | .14*** | -.13*** | .06* | .01 | -.03 |
| 8. | Independence (w8)^1^ |  |  |  |  |  |  | 1 | -.30*** | -.03 | -.02 | -.04 | .05* | .13*** | -.02 | -.05+ | .03 |
| 9. | Struggling of the child (w9) |  |  |  |  |  |  |  | 1 | .09*** | .09** | .07** | -.11*** | -.04 | -.02 | -.04 | .01 |
| 10. | Children under 14 (w9) |  |  |  |  |  |  |  |  | 1 | .92*** | .12*** | -.15*** | -.09*** | .002 | .11*** | -.27*** |
| 11. | Children under 14 (w8) |  |  |  |  |  |  |  |  |  | 1 | .12*** | -.04 | -.10*** | .01 | .01 | -.27*** |
| 12. | Perceived control (w9) |  |  |  |  |  |  |  |  |  |  | 1 | -.11*** | .02 | .01 | .22*** | -.18*** |
| 13. | Age respondent (w8) |  |  |  |  |  |  |  |  |  |  |  | 1 | .02 | .02 | .05* | .10*** |
| 14. | Sex respondent |  |  |  |  |  |  |  |  |  |  |  |  | 1 | -.01 | .20*** | -.04 |
| 15. | Sex child |  |  |  |  |  |  |  |  |  |  |  |  |  | 1 | -.13*** | .08** |
| 16. | Childcare situation (w9) |  |  |  |  |  |  |  |  |  |  |  |  |  |  | 1 | -.20*** |
| 17. | Employment situation (w9) |  |  |  |  |  |  |  |  |  |  |  |  |  |  |  | 1 |

Note. SES, socioeconomic status, highest ISEI of family. HLE, Home learning environment. Independence, School-related independence. w7, wave 7. w8, wave 8. w9, wave 9. ^1^Mean of indicators. ^2^Mean of standardized indicators. Correlations estimated with weighted data. ^+^*p*< 0.10^*^*p*< 0.05, ^**^*p*< 0.01, ^***^*p*< 0.001.

## Structural equation model of dealing with the pandemic

*Supplementary Table 3.* Predictors and Covariances in Main Model (with weighted and unweighted data).

| **Constructs** | **Predictors** | **Coefficient (std)/ SE/ P>\|t\|** | |
| --- | --- | --- | --- |
|  |  | weighted | unweighted |
| Self-assessed supporting abilities; w9 | Home learning environment; w8  School-related independence; w8  SES (HISEI); w7  Education respondent in years; w7  Sex child (1 = female; 2= male)  Employment respondent before lockdown; w9  Childcare situation; w9  Age respondent  Sex respondent  Children under 14; w9  Perceived control; w9  Psychological stress; w7  Self-regulation child; w7 | -0.016/ 0.089/ 0.862  -0.052/ 0.100/ 0.607  0.031/ 0.087/ 0.722  0.313/ 0.098/ 0.001  -0.114/ 0.061/ 0.062  -0.026/ 0.065/ 0.688  -0.055/ 0.070/ 0.430  -0.025/ 0.069/ 0.716  0.164/ 0.087/ 0.060  -0.144/ 0.076/ 0.057  0.007/ 0.071/ 0.915  -0.213/ 0.097/ 0.028  -0.040/ 0.111/ 0.715 | -0.039/ 0.030/ 0.198  0.092/ 029/ 0.001  0.100/ 0.029/ 0.001  0.144/ 0.029/ 0.000  -0.045/ 0.023/ 0.052  -0.011/ 0.024/ 630  0.018/ 0.023/ 0.426  0.011/ 0.024/ 0.641  0.007/ 0.022/ 0.748  -0.023/ 0.024/ 0.336  0.020/ 0.023/ 0.372  -0.155/0.032/ 0.000  -0.003/ 0.031/ 0.906 |
| Perceived stress; w9 | Home learning environment; w8  School-related independence; w8  SES (HISEI); w7  Education respondent in years; w7  Sex child (1 = female; 2= male)  Employment respondent before lockdown; w9  Childcare situation; w9  Age respondent  Sex respondent  Children under 14; w9  Perceived control; w9  Psychological stress; w7  Self-regulation child; w7 | 0.020/ 0.096/ 0.835  -0.166/ 0.108/ 0.127  -0.226/ 0.084/ 0.007  0.147/ 0.072/ 0.043  0.079/ 0.073/ 0.281  0.135/ 0.073/ 0.065  0.110/0. 070/ 0.116  0.092/ 0.082/ 0.262  0.039/ 0.044/ 0.371  -0.081/ 0.071/ 0.254  0.034/ 0.076/ 0.649  0.289/ 0.116/ 0.013  0.005/ 0.091/ 0.954 | -0.021/ 0.028/ 0.458  -0.101/ 0.028/ 0.000  0.008/ 0.029/ 0.765  -0.022/ 0.028/ 0.443  -0.019/ 0.022/ 0.401  0.084/ 0.022/ 0.000  0.078/ 0.021/ 0.000  0.001/ 0.022/ 0.964  0.048/ 0.021/ 0.021  0.075/ 0.023/ 0.002  0.223/ 0.020/ 0.000  0.225/ 0.031/ 0.000  -0.054/ 0.030/ 0.076 |
| Child struggling with homeschooling; w9 | Home learning environment; w8  School-related independence; w8  SES (HISEI); w7  Education respondent in years; w7  Sex child (1 = female; 2= male)  Children under 14; w9  Psychological stress; w7  Self-regulation child; w7 | 0.019/ 0.084/ 0.818  -0.272/ 0.078/ 0.001  0.172/ 0.081/ 0.036  -0.016/ 0.079/ 0.834  -0.039/ 0.062/ 0.528  0.120/ 0.067/ 0.076  0.297/ 0.076/ 0.000  0.043/ 0.088/ 0.625 | -0.022/ 0.029/ 0.447  -0.233/ 0.028/ 0.000  0.040/ 0.029/ 0.176  -0.060/ 0.029/ 0.038  -0.025/ 0.023/ 0.273  0.045/ 0.023/ 0.049  0.176/ 0.032/ 0.000  -0.012/ 0.031/ 0.702 |
| Home learning environment; w8 | SES (HISEI); w7  Education respondent in years; w7  Sex child (1 = female; 2= male)  Children under 14; w8  Psychological stress; w7  Self-regulation child; w7 | 0.200/ 0.098/ 0.042  -0.054/ 0.098/ 0.578  -0.237/ 0.086/ 0.006  0.029/ 0.126/ 0.813  -0.122/ 0.137/ 0.373  0.003/ 0.153/ 0.982 | 0.148/ 0.037/ 0.000  0.005/ 0.037/ 0.873  -0.105/ 0.030/ 0.000  -0.105/ 0.029/ 0.612  -0.072/ 0.041/ 0.076  0.070/ 0.038/ 0.066 |
| School-related independence; w8 | SES (HISEI); w7  Sex child (1 = female; 2= male)  Self-regulation child; w7 | 0.284/ 0.077/ 0.000  -0.038/ 0.103/ 0.707  0.229/ 0.146/ 0.117 | 0.182/ 0.027/ 0.000  0.070/ 0.027/ 0.010  0.225/ 0.032/ 0.000 |
| **Covariance** | |  |  |
| Self-assessed supporting abilities, perceived stress | | -0.082/ 0.070/ 0.239 | -0.143/ 0.024/ 0.000 |
| Self-assessed supporting abilities, child struggling with homeschooling | | -0.262/ 0.074/ 0.000 | -0.186/ 0.023/ 0.000 |
| Perceived stress, child struggling with homeschooling | | 0.170/ 0.064/ 0.008 | 0.340/ 0.022/ 0.000 |
| SES (HISEI); w7, education respondent in years; w7 | | 0.560/ 0.0507 0.000 | 0.590/ 0.015/ 0.000 |
| SES (HISEI); w7, psychological stress; w7 | | 0.059/ 0.112/ 0.596 | -0.068/ 0.030/ 0.026 |
| SES (HISEI); w7, self-regulation child; w7 | | -0.217/ 0.088/ 0.014 | -0.030/ 0.028/ 0.289 |
| Education respondent in years; w7, psychological stress; w7 | | -0.049/ 0.108/ 0.650 | -0.050/ 0.030/ 0.101 |
| Education respondent in years; w7, self-regulation child; w7 | | -0.076/ 0.083/ 0.358 | -0.029/ 0.027/ 0.290 |
| Psychological Stress; w7, self-regulation child; w7 | | -0.336/ 0.096/ 0.000 | 0.033/ 0.030/ 0.273 |
| Note. SES, socioeconomic status, highest ISEI of family. . w7, wave 7. w8, wave 8. w9, wave 9. Estimated with weighted data. Measurement models of latent variables in the supplement in Table 1. Model fit of estimation with unweighted data: N = 1,812; Chi² (248) = 643.76, CFI = 0.93, TLI = 0.91, RMSEA = 0.03. | | | |


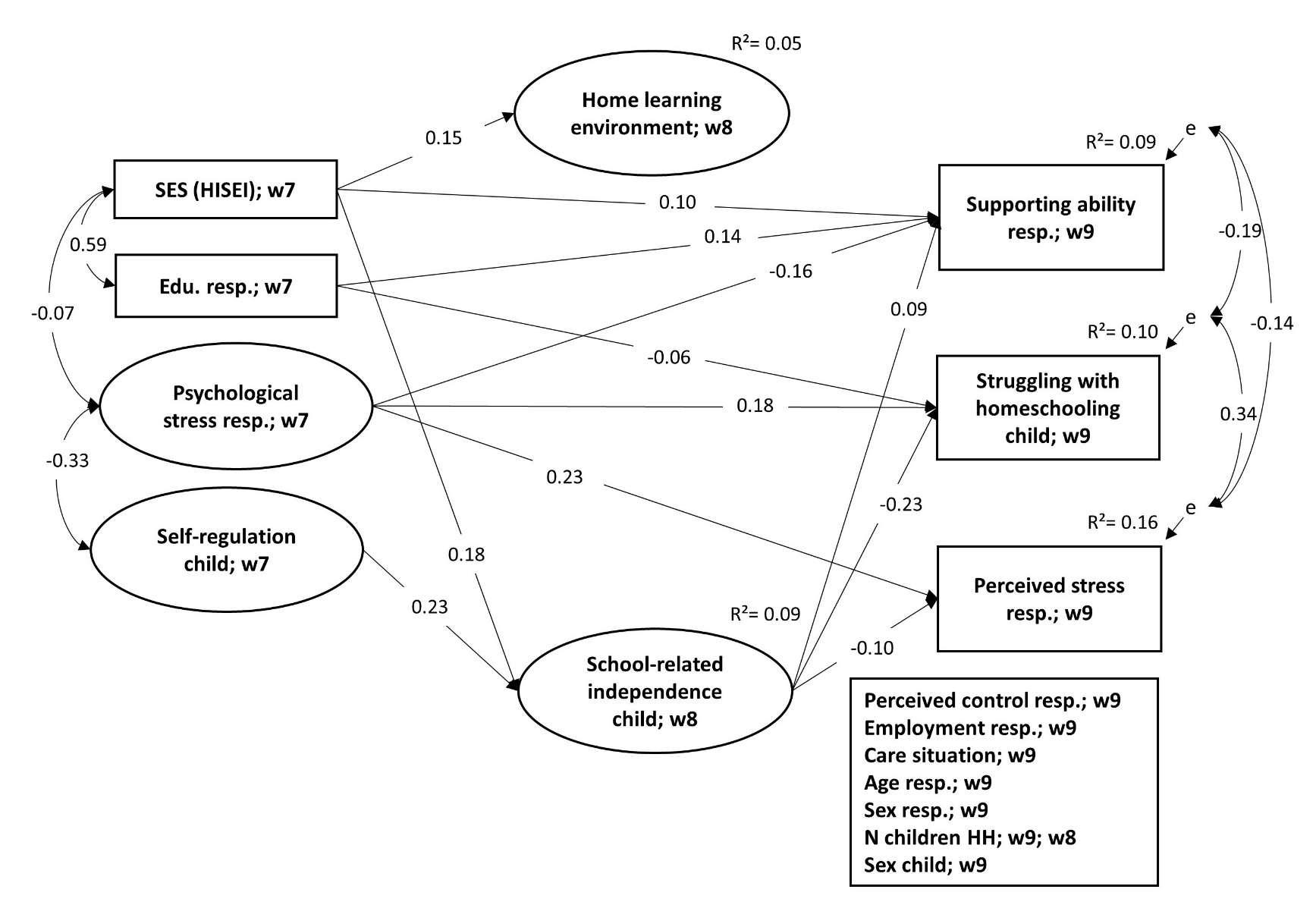


*Supplementary Figure 1*. Results of the structural equation model for self-assessed supporting ability and perceived stress during the school closure estimated with unweighted data (algorithm mlmv). Only the significant standardized coefficients are presented (p < 0.05). SES, socioeconomic status, highest ISEI of the family. Edu. resp., education of the respondent in years. w, wave. Measurement models of latent variables in the supplement in Table 1. Model fit: N = 1,812; Chi² (248) = 643.76, CFI = 0.93, TLI = 0.91, RMSEA = 0.03.

## Additional models with migration background

Our additional model (see Figure 2 in the Supplementary Material) showed only one significant effect of migration background, which affected the home learning environment positively. In other words, families with a migration background reported a higher amount of stimulating activities with their child, when controlling for the SES of the family and the education of the respondent. All other associations with migration background (besides covariations with the structural factors) were not significant. The additional model replicated the results of the main model with the exception of one effect. In the additional model, the years of education of the responding parent did not significantly predict perceived stress but did predict the parent’s ability to provide support. Apart from that, the fit of the additional model that included migration background (estimated with unweighted data; model fit calculated with unweighted data; for the model with unweighted data see Figure 3 in the Supplementary Material) was deteriorating, below an acceptable fit for the TLI (0.89).


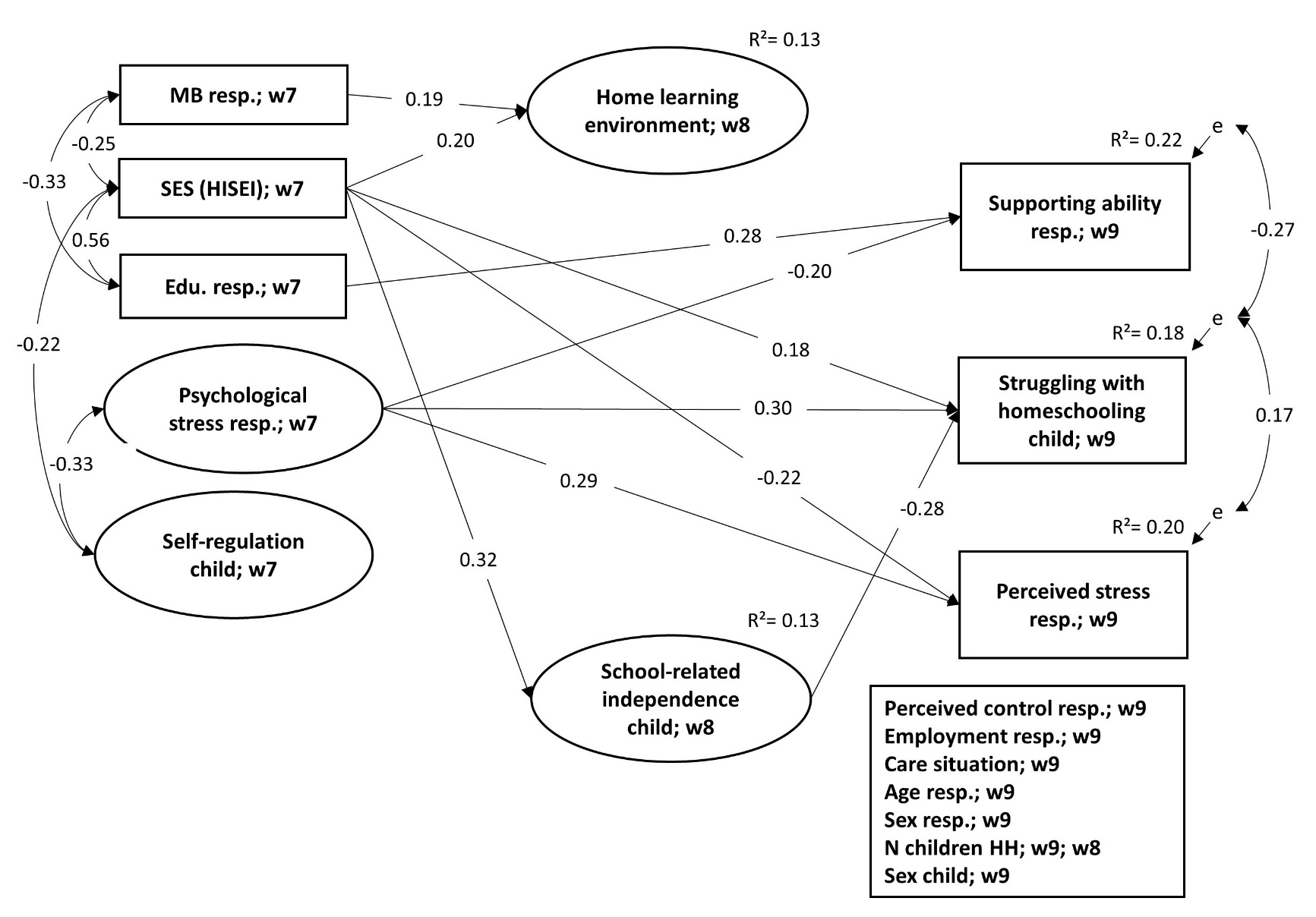


*Supplementary Figure 2*. Results of the structural equation model for self-assessed supporting ability and perceived stress during school closure including migration background of respondent as predictor (algorithm mlmv). Only the significant standardized coefficients are presented (p < 0.05). SES, socioeconomic status, highest ISEI of the family. Edu. resp., education of the respondent in years. MB resp., migration background of the respondent. w, wave. Estimated with weighted data. Measurement models of latent variables in the supplement in Table 1. Model fit of estimation with unweighted data: N = 1,812; Chi² (259) = 722.09, CFI = 0.92, TLI = 0.89, RMSEA = 0.03.


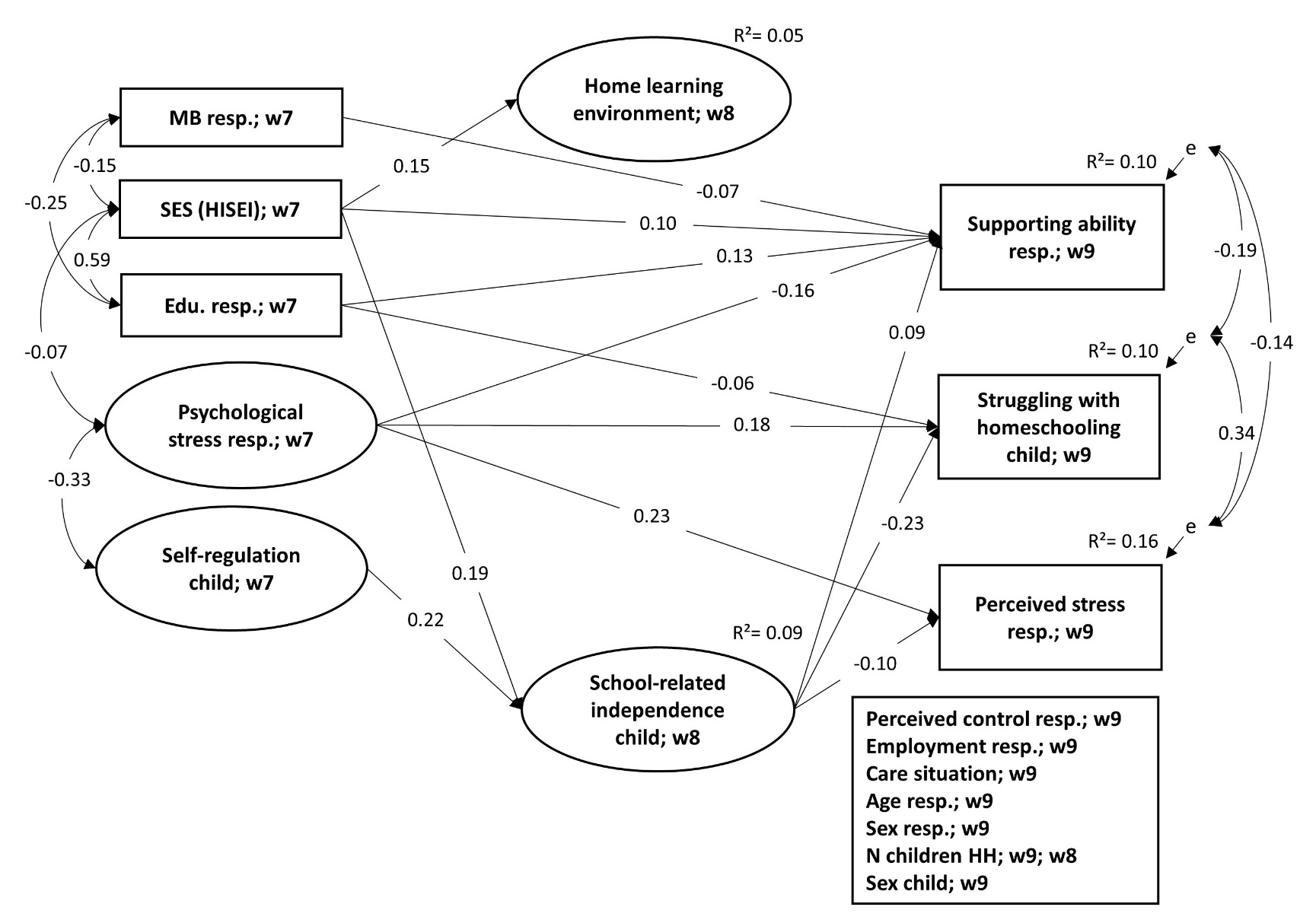


*Supplementary Figure 3*. Results of the structural equation model for self-assessed supporting ability and perceived stress during the school closure including migration background of respondent as predictor estimated with unweighted data (algorithm mlmv). Only the significant standardized coefficients are presented (p < 0.05). SES, socioeconomic status, highest ISEI of the family. Edu. resp., education of the respondent in years. MB resp., migration background of the respondent. w, wave. Measurement models of latent variables in the supplement in Table 1. Model fit of estimation: N = 1,812; Chi² (259) = 711.09, CFI = 0.92, TLI = 0.89, RMSEA = 0.03.
